# Optimizing multiprocessor performance in real-time systems using an innovative genetic algorithm approach

**DOI:** 10.1038/s41598-024-80910-4

**Published:** 2025-01-30

**Authors:** Heba E. Hassan, Khaled Hosny Ibrahiem, Ahmed H. Madian

**Affiliations:** 1 Department of Electrical Engineering, Faculty of Engineering, Fayoum University, Fayoum, Egypt; 2 Nanoelectronics Integrated Systems Center (NISC), Nile University, Giza, 12677 Egypt; 3 Radiation Engineering Department, Nuclear Research Center (NRC), Egyptian Atomic Energy Authority, Cairo, 13759 Egypt

**Keywords:** Multiprocessors, Task Scheduling, Genetic algorithms, Performance utilization, Multiprocessor, No-Preemptions, Computational science, Computer science

## Abstract

Due to its enormous influence on system functionality, researchers are presently looking into the issue of task scheduling on multiprocessors. Establishing the most advantageous schedules is often regarded as a difficult-to-compute issue. Genetic Algorithm is a recent tool employed by researchers to optimize scheduling tasks and boost performance, although this field of research is yet mostly unexplored. In this article, a novel approach for generating task schedules for real-time systems utilizing a Genetic Algorithm is proposed. The approach seeks to design task schedules for multiprocessor systems with optimal or suboptimal lengths, with the ultimate goal of achieving high performance. This research project focuses on non-preemptive independent tasks in a multiprocessor environment. All processors are assumed to be identical. We conducted a thorough analysis of the proposed approach and pitted it against three frequently utilized scheduling methodologies: the “Evolutionary Fuzzy Based Scheduling Algorithm”, the “Least Laxity First Algorithm”, and the “Earliest Deadline First Algorithm”. The Proposed Algorithm demonstrated superior efficiency and reliability compared to Earliest Deadline First, Least Laxity First, and Evolutionary Fuzzy-based Scheduling Algorithm. It consistently achieved zero missed deadlines and the lowest average response and turnaround times across all scenarios, maintaining optimal performance even under high load conditions.

## Introduction

Computer systems with a primary function to fulfill tasks on time are called Real-time systems. All of the tasks in these systems have parameters that include arrival time, execution time, deadline, and priority. The most recent embedded computing systems are growing increasingly complicated. Real-time systems are distinctly divided into two categories, namely “Soft” and “Hard” real-time systems. Failure to fulfill a deadline is seen as a serious issue in “hard” real-time systems. In contrast, soft real-time systems readily permit it. In real-time systems, scheduling approaches are required to meet tight timing requirements and ensure a system’s faithful and predictable performance.

A variety of scheduling algorithms are used in multiprocessor scheduling, even though the majority are usually implemented on uniprocessor systems. “Least Laxity First (LLF)” is a widely recognized algorithm that selects and executes the task with minimal Laxity. If two tasks have the same laxities, the preemptive form of LLF may encounter several context switches. One more famous scheduling algorithm is “Earliest Deadline First” (EDF)^1,2^, The system confidently prioritizes the task with the soonest deadline to be executed. Both preemptive and non-preemptive EDF strategies are possible. Both approaches may be utilized on platforms with one or more processors and are dynamic. They are regarded as optimal for uniprocessor platforms, but nevertheless, they fail in overloaded scenarios.

Various advancements in traditional algorithms have been introduced. One of these is the: “Fixed-Priority till Zero Laxity (FPZL)”^3^ approach, which maintains the task’s priority until the laxity of it reaches zero. The aforementioned algorithm, together with “Fixed Priority until Critical Laxity (FPCL)” and “Fixed Priority until Static Laxity (FPSL):^4^, are considered minimal dynamic scheduling methods for limiting the number of preemptions. Another approach can be “Earliest Deadline until Zero Laxity (EDZL)”^5,6^in which EDF alongside LLF are combined. obtaining the optimum schedule regarding a collection of tasks is rated NP-hard^2–7^.

Fuzzy logic may also be employed to solve the scheduling problem. “An Evolutionary Fuzzy Based Scheduling Algorithm (EFSBA) " was suggested by Hassan, Heba E., et al.^2^. In order to create task schedules, this approach incorporates two inference engines. This approach is restricted to independent tasks that are not preemptive in a multiprocessor system.

Heuristics and meta-heuristic approaches or hybrids of meta-heuristics and other techniques, such as heuristics and machine learning, are some of the several types of task scheduling approaches. Heuristic-based algorithms produce solutions close to the optimum for a given problem. The meta-heuristics techniques, on the other hand, are specially built for generalized optimum solutions that may be used across various domains^8–12^.

Task scheduling approaches incorporating meta-heuristics are classified as evolutionary-based, such as “Genetic Algorithm”^13^, “bio-meta-heuristics”, and “non-bio-meta-heuristics”, such as “Simulation-Based Optimization (SBO)” and “Simulated Annealing (SA)”^14^. A branch of computational intelligence called swarm intelligence (SI)^15^ pioneered by^16^. Swarm Intelligence seeks to address computational issues by Simulating autonomously organized groupings of items interacting with one another. By exchanging information, agents may share their experiences^17^. Meta Performance Effective Genetic Algorithm (PEGA) has been introduced by Ahmad, S. G., et al.^18^. The PEGA algorithm efficiently selects the best solution from the space for investigation. It achieves high performance by implementing effective crossover and mutation operators.

In their publication^19^, A. Sharma et al. developed a height-based genetic algorithm (GA) to solve scheduling problems for dependent tasks in a multiprocessor architecture. The algorithm assigns task priorities based on their height, number of children, and execution time. Experimental simulations compared this approach with a Basic GA using various task graphs and numbers of generations. In a study published in^20^, Agarwal et al. proposed a Neurogenetic metaheuristic approach that combines an improved neural network with a genetic algorithm. The outcomes reveal that when employed independently, the Neurogenetic approach outperforms both the upgraded neural network and the genetic algorithms.

Hegde, S.N., et al. in^21^ has created an advanced multi-objective task scheduling framework tailored specifically for heterogeneous grid networks. This innovative framework aims to significantly minimize turnaround time, communication overhead, and execution costs, all while maximizing the overall utilization of the grid system. In their publication^22^, Chauhan, N., et al. proposed a model utilizing two hybrid genetic algorithms (HHCGA and HHAGA) to solve this NP-hard issue. This model aimed to optimize system cost, response time, and system reliability. This model is applicable to various real-life systems like transportation, telecommunication, and banking systems. In their study cited as^23^, A cutting-edge hybrid metaheuristic approach has been meticulously developed. This algorithm utilizes a greedy randomized adaptive search procedure to construct high-quality solutions, which are then further enhanced using a genetic algorithm. The algorithm is driven by two heuristic functions, namely bottom-level and top-level, and has undergone rigorous testing using benchmark test problems.

In^24^ Kumar, H., et al. introduced a task allocation strategy for Distributed Real-Time Systems (DRTS). This paper presented a new model for clustering tasks and assigning them to processors based on two distance measures: Yang’s distance and Hamming’s distance This approach is aimed at balancing the load across processors and minimizing communication overhead, thereby improving the overall efficiency of DRTS. The authors in^25^ addressed two main approaches: heuristic-based priority assignment and Audsley’s optimal priority assignment (OPA) algorithm, which is limited to certain schedulability analyses. proposed a novel priority assignment framework combining the strengths of both approaches. The framework and hybrid algorithm are more effective at scheduling task sets than previous methods, particularly when higher accuracy is required for schedulability analysis.

A novel task scheduling method using a hybrid Particle Swarm Optimization (PSO) technique was introduced in^26^. The objective of this method is to address task allocation problems in heterogeneous distributed computing systems by optimizing system cost, response time, and flow time. This method is suitable for real-world applications in distributed systems where task allocation is critical for performance optimization. The study^27^ proposed the Hybrid Vulture Ant Lion Model (HV-ALM), which combines the strengths of vulture and ant lion optimization algorithms. This model improves the efficiency of task scheduling by reducing execution time, makespan, memory use, and power consumption compared to traditional models. The HV-ALM outperforms existing models in several metrics, including reduced memory usage (30%), makespan (450 s), and power consumption (1.5 mW). Table 1 categorizes some recent research, providing a concise overview of advancements in task scheduling strategies during the last few years.

**Table 1:** Overview of literature on task scheduling in distributed systems.

S. No.	Ref.	Year	Authors	Method classification	Application domain
1	^1^	2019	Heba E. Hassan, Gihan Nagib, Khaled Hosny Ibrahiem	Evolutionary Fuzzy-Based Scheduling Algorithm (EFBSA)	Soft Real-Time Multiprocessor Systems
2	^7^	2021	Heba E. Hassan, Gihan Nagib, Khaled Hosny Ibrahiem	Priority-Fuzzy-B-Level (PFB) algorithm, based on fuzzy logic and task priority parameters	Dependent Non-Preemptive Task Scheduling on Homogeneous Multiprocessor Systems
3	^8^	2022	Said Nabi, Masroor Ahmad, Muhammad Ibrahim, Habib Hamam	Adaptive Particle Swarm Optimization (AdPSO)	Cloud Computing
4	^9^	2018	R. Logesh, V. Vijayakumar V. Subramaniyaswamy, D. Malathi, N. Sivaramakrishnan,	Bio-inspired Clustering Ensemble (BICE)	Recommender Systems
5	^10^	2018	R. Logesh, V. Indragandhi V. Subramaniyaswamy, V. Vijayakumar, Xiao-Zhi Gao,	Quantum-behaved Particle Swarm Optimization (QPSO) and Clustering Ensemble (QICE)	Recommender Systems for Urban Trip Planning in Smart Cities
6	^11^	2021	Aroosa Mubeen, Muhammad Ibrahim, Nargis Bibi, Mohammed Baz, Habib Hamam, Omar Cheikhrouhou	Adaptive Load Balanced Task Scheduling (ALTS) integrating GA and ACO algorithms	Cloud Computing Task Scheduling for Optimized Load and SLA Compliance
7	^17^	2018	Okkes Ertenlice, Can B. Kalayci	reviews the use of Swarm Intelligence Algorithms (e.g., Particle Swarm Optimization, Artificial Bee Colony)	Portfolio Optimization​
8	^21^	2024	Sujay N. Hegde, D. B. Srinivas, M. A. Rajan, Sita Rani, Aman Kataria, Hong Min	Multi-objective Optimization with Greedy Scheduling Variants (e.g., TOPSIS, GridSim Simulation)	Task Scheduling in Computational Grids
9	^22^	2022	Nutan Kumari Chauhan, Isha Tyagi, Harendra Kumar, Dipa Sharma	Hybrid Genetic Algorithm (HHCGA and HHAGA) combining hierarchical clustering and heuristic approach	Real-Time Systems on Heterogeneous Multiprocessor Environments
10	^24^	2020	Kumar, Tyagi	Modified Hierarchical Clustering (MHC)	Task scheduling in Distributed Real-Time Systems (DRTS)
11	^25^	2024	Xuanliang Deng, Shriram Raja, Yecheng Zhao, Haibo Zeng	Hybrid Priority Assignment Algorithm combining MITER and DkC for Global Fixed-Priority Scheduling	Real-Time Systems on Multiprocessor Platforms
12	^26^	2023	Karishma, H. Kumar	Hybrid Particle Swarm Optimization (PSO)	Task scheduling in distributed computing systems
13	^27^	2024	Naga Deepa Choppakatla, M. K. Chaitanya Sivalenka, Ravi Boda	Hybrid Vulture Ant Lion Model (HV-ALM)	Multiprocessor Embedded Systems
14	^30^	2023	Tao Hai, Jincheng Zhou, Dayang Jawawi, Dan Wang, Uzoma Oduah, Cresantus Biamba, Sanjiv Kumar Jain	HEFT (Heterogeneous Earliest Finish Time) and Altered HEFT Versions (MXCT, MNCT, AVBS)	Task Scheduling in Cloud Computing​

In this article, the Optimized Performance Based Genetic Algorithm (OPBGA) for multiprocessors with soft real-time nature is introduced to schedule independent tasks that are not preemptive. The purpose of this algorithm is to discover task schedules that have either optimal or suboptimal durations, leading to improved performance for multiprocessor systems. We compared our approach to other algorithms, such as the “Evolutionary Fuzzy-based Scheduling Algorithm (EFSBA)”, the “Least Laxity First (LLF)” algorithm, and the “Earliest Deadline First (EDF)” algorithm. Our comparison considers the “Average Turnaround Time (ATAT)”, “Average Response Time (ART)”, and the amount of “Deadline Misses (DLMs)”. All processors used in our experiment are identical and constant in number. Our proposed technique demonstrated significantly higher accuracy and speed compared to previous approaches.

## Proposed algorithm

Genetic Algorithm (GA) is being utilized to solve optimization issues in an effective manner. The GA effectively creates the advantage of global spaces for seeking the best and most advantageous solutions to the issue. In a similar way, GA operators like crossover and mutation can be modified to make them more appropriate to the problem at hand. Similarly, the initial population’s composition of various arrangements has a broad impact on the overall exhibition^28–30^. In this paper, an innovative GA-based technique for scheduling task on multiprocessor in real-time environment will be provided. The population is produced by allocating and scheduling tasks to random processors. To produce offspring, the best chromosome is chosen through the evaluation of its fitness value via crossover and mutation operators.

### Gene representation

To represent the genes of chromosomes, we used decimal integers. each chromosome is produced in a random way with two parts: “Tasks” and “Processors”. The “tasks” component provides clear guidance on the necessary sequence for completing tasks, whereas the processors section includes processor indices where the tasks will be conducted. Suppose there are n available tasks. Consequently, the length of the chromosome is now “2n”, or twice as many accessible tasks.

**Fig. 1:**
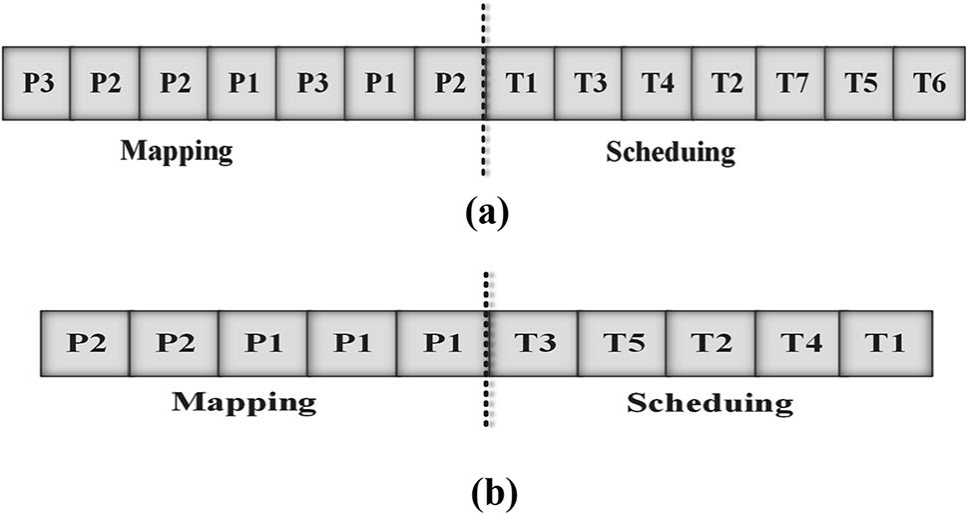
(**a**) The chromosome structure (**b**) A typical chromosome for the case study task set generated by the proposed algorithm.

The components in the Processors section are generated at random from “1” up to “m”, as the total number of available processors is denoted by the letter “m”. Assuming a real-time system with three processors, thus, “m” equals 3. The Tasks component is also randomly selected. Then, as illustrated in Fig. 1, the Processor and Tasks components are combined to form the whole chromosome. Figure 1(a) shows that task T1 is assigned to processor P3, task T3 to processor P2, task T4 to processor P2, and so on. A representative chromosome produced by our algorithm is shown in Fig. 1(b).

### Initialization of the population

Every chromosome in the population represents a potential solution. In order to create the first population, a particular set of chromosomes is created randomly. This collection of chromosomes stands for a variety of solutions that serve in scanning the searching space.

### Fitness function and assessment of fitness value

The primary goal is to shorten the duration and decrease the delay of completing all tasks. To achieve this, a schedule needs to be created for “n” tasks to be done on “m” processors in the least amount of time possible. The quality of the solution is directly affected by the length of the schedule.

That could be accomplished by reducing the length of the schedule, the number of Deadlines Missed (DLM), the Average Response Time (ART), and the Average Turnaround Time (ATAT)^1,31,32^. Whereas, in soft real-time environments, it is acceptable to miss some deadlines even though DLM is a statistic used to measure tasks that fail to meet their deadlines. The measurement known as Response Time (RT) indicates the amount of time it takes to deliver the initial response following a request, as explained in sources^1,32^. Calculating ART is a straightforward process that involves adding up the response time for each task and dividing it by the total number of tasks. The term Turnaround Time (TAT) refers to the duration it takes for a task to be finished from its submission moment. To calculate ATAT, you need to add up the TATs for all tasks and then divide that sum by the total number of tasks^1,31,32^.

When assessing how well scheduling algorithms operate, the number of DLMs is a crucial consideration. So, the amount of DLMs is a key factor in evaluating the fitness value. The pseudocode of the fitness function is shown below.
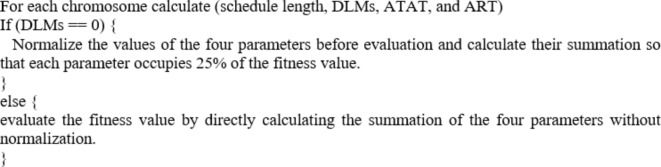


The main reason for evaluating fitness value in this manner is to ensure that chromosomes with zero deadline misses have a great chance to survive. then, if they survive, it selects the chromosome with the highest performance based on the other three characteristics (i.e., schedule length, ART, and ATAT). If no chromosome in the search space meets the criterion of zero deadline misses, the second part of the fitness function will assign the best fitness value to the chromosome with the minimum DLMs.

For more clarification, the first part of the fitness function (zero DLMs) produces fitness values less than 0.75 due to the normalization step, whereas, on the other hand, the second part of the fitness function (nonzero DLMs) gives fitness values greater than 1. That will help getting rid of chromosomes that have deadline misses or minimizing the DLMs if exist, while also ensuring high performance. After determining fitness, the ones having the highest possible fitness scores are selected.

### Selection

The algorithm starts by randomly generating the initial population. Afterward, a series of new populations are produced by the algorithm. Then it creates the next population using individuals from the current generation at each step. In order to develop the new population, the algorithm assesses each individual member within the existing population by the assessment of its fitness value, these values are referred to as “raw fitness scores”. These scores are subsequently rated to produce an extra meaningful set of outcomes. Such rated scores are called “expectation values”.

The algorithm then chooses members, known as “parents”, based on their expectations. On the other hand, some of the current population’s less fit individuals are selected as an “elite”. These “elite” individuals are passed along to the next generation. By randomly altering the vector entries of a single parent, “Children " are created from “parents” (through mutation) or by mixing vector entries of two parents (crossover) as specified later. The MATLAB’s @selectionstochunif, the default selection option, is used in for choosing the “parents” in the proposed algorithm, which draws a line with each parent denoting a section of the line whose length is proportional to its scaled value.

### Reproduction operators

In order to produce the offspring that will make up the following generation, the population is employed by the genetic algorithm throughout every stage. From the current population, GA selects a group of individuals, known as parents, who pass on their genes, the entries in their vectors, to their children. Typically, the system chooses parents who have higher fitness ratings. In the proposed approach, the crossover and mutation functions used are designed as illustrated below.

#### Crossover function

**Fig. 2:**
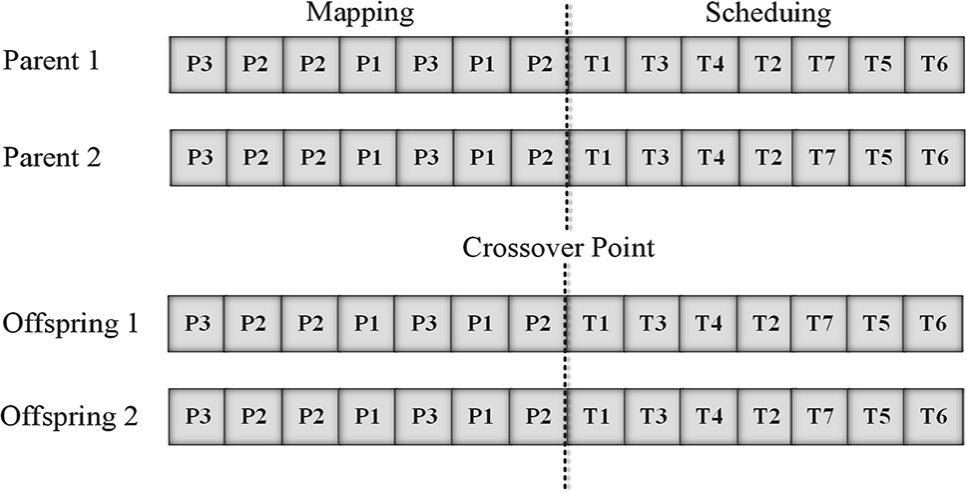
Crossover operation.

As was previously stated, chromosomes are composed of two parts: tasks and processors. Two chromosomes are picked from the population and then undergo a single-point crossover. The crossover point is where the two parts of the chromosome meet. After then, two new offspring are produced by swapping the chromosomal parts to the right of the “crossover point”. In order to demonstrate the variations in processor assignments prior to and following a crossover operation, Fig. 2 depicts a one-point crossover.

#### Mutation function

**Fig. 3:**
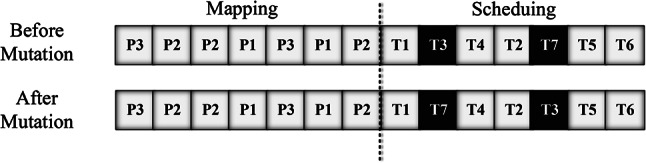
Mutation operation.

Every time a mutation occurs, one chromosome is chosen at random. Any point in the “tasks” part is expected to undergo a mutation operation. Figure 3 depicts a chromosomal random mutation procedure. It switches the assignment of two tasks. This is accomplished by randomly switching two tasks from the “tasks” part of the chromosome. Following the mutation operation, task TX is allocated to processor TY, while task TY is assigned to processor TX.

### Case study

This section provides a simple example that demonstrates the concept. Five real-time tasks and two processors were used in this example. The parameters of the jobs are shown in Table 2, including their arrival time, execution time, deadline, and laxity respectively.

**Table 2:** A task set is made up of five tasks.

Task ID	Arrival time	Computation time	Deadline	Laxity
1	0	7	14	7
2	0	3	7	4
3	0	5	17	12
4	0	4	19	15
5	0	8	15	7

**Fig. 4:**
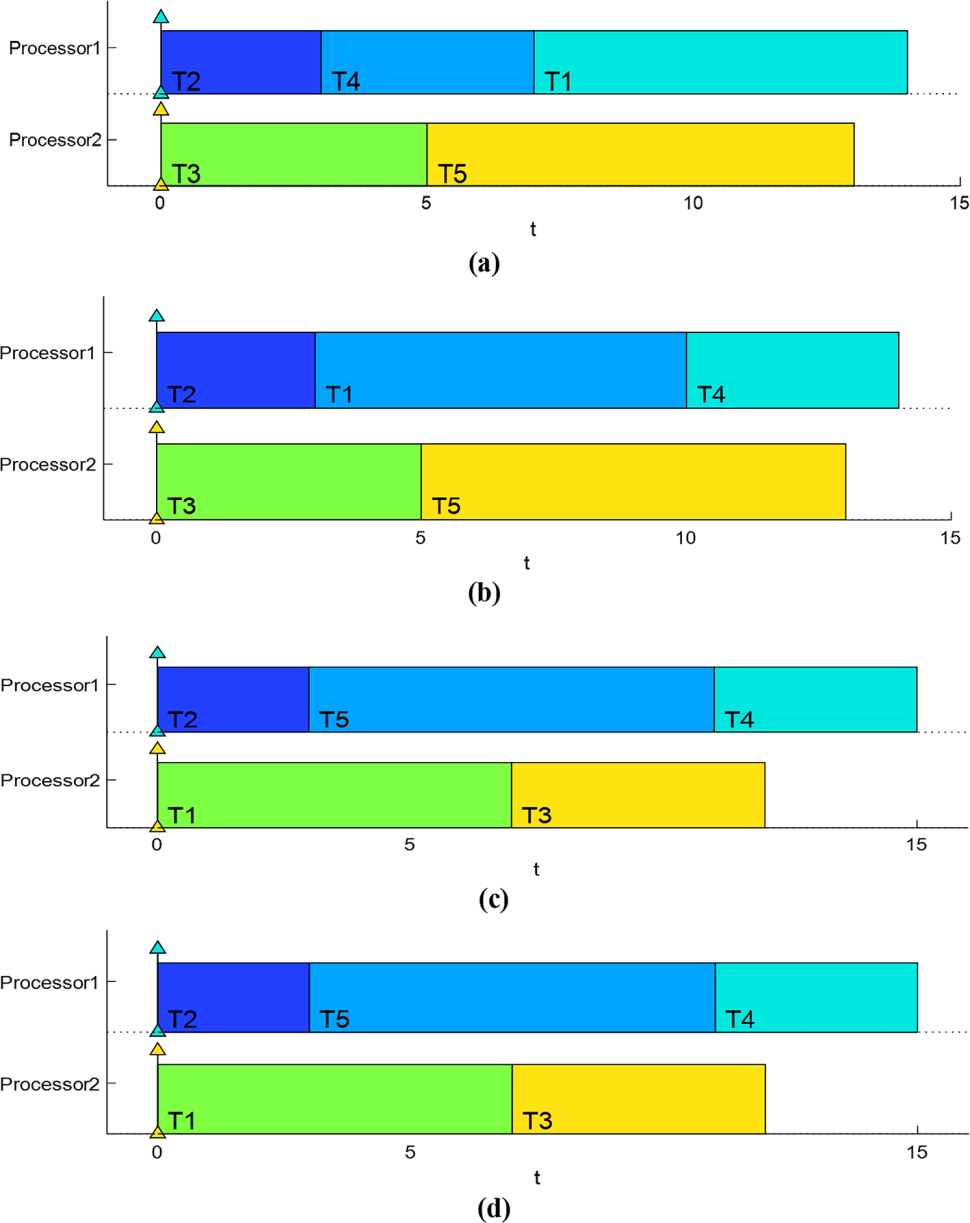
Gantt chart of the mentioned case study calculated by (**a**) optimized performance based genetic algorithm (OPBGA) (**b**) evolutionary fuzzy-based scheduling algorithm (EFSBA) (**c**) earliest deadline first (EDF) algorithm (**d**) least laxity first (LLF) algorithm.

The previously mentioned task set has a load factor (U) of 1.966548725 which is assigned to two processors, m = 2. Thus, the ratio of U/m obtained is equal to 0.98325 where, the utilization bound is 0.743492. Figure 4 shows the time schedules generated by each of the four algorithms, while Table 3 provides various performance evaluations according to DLMs, ART, and ATAT parameters.

**Table 3:** Performance measures of the four algorisms.

Algorithm	DLMs	ART	ATAT
OPBGA	0	3	8.4
EFSBA	0	3.6	9
LLF	0	4.2	9.6
EDF	0	4.2	9.6

The Gantt charts in Fig. 4 illustrate that the 14 u.t. schedule lengths introduced by the OPBGA and EFSBA algorithms are superior than the 15 u.t. schedule lengths generated by the LLF and EDF algorithms. On the other hand, Table 3 shows that OPBGA algorithm outperformed the other algorithms with the least ART and ATAT.

## Performance assessment and results

The MATLAB simulator was employed for implementing the suggested method. The recommended algorithm’s functionality was assessed in comparison to “EFSBA”, “LLF”, and “EDF”. This comparison was built using carefully chosen measures to exactly analyze the performance of different algorithms. The “DLM”, “ART”, “ATAT” are among the performance aspects evaluated^1,31,32^.

The computational complexities of the aforementioned methods are contingent upon their respective designs and operational requisites. Earliest Deadline First (EDF) exhibits a complexity of O (n log n) due to the requisite task sorting by deadlines. In contrast, Least Laxity First (LLF) is characterized by a more computationally intensive complexity of O(n^2) owing to the frequent recalculation of laxities for all tasks. Fuzzy logic scheduling approaches, such as EFSBA, are contingent upon the quantity of inputs and rules, with a complexity of O (n + m), where n represents the number of inputs and m denotes the number of rules. This delineates its general efficiency while highlighting its reliance on system size. Lastly, methodologies employing Genetic Algorithms (GAs), exemplified by the proposed algorithm ‘OPBGA’, demonstrate a higher complexity of O (G × P × L), where G signifies the number of generations, P represents the population size, and L denotes the chromosome length. This attributes effectiveness in tackling large, intricate problems, albeit at the cost of heightened computational intensity.

The datasets analyzed and generated during this study are not available on any online platform. They were created using MATLAB, as mentioned in the manuscript, and any required portion can be obtained from the corresponding author upon reasonable request. Moreover, the authors are preparing a paper that will offer a benchmarking perspective on these research results. This paper will provide other researchers with a comprehensive understanding of this topic.

Different numbers of processors can be used to implement the suggested method. Nonetheless, the evaluation for comparison involved different scenarios of using three, ten, and one hundred processors. Considering each scenario of, randomized task sets were originated in order to fully assess the proposed approach. These task sets originated using uniform distribution along with additional statistical computations so as to adequately address every single problem possibility.

For the three processors scenario, Table 4, task sets were randomly generated with a range of 5 to 110 tasks and load factor (U) values that varied between 0.12 and 3. The range of laxities in this situation was from zero to ten-time units, whereas the deadlines varied between one- and twenty-time units.

**Table 4:** Performance measures in the case of 3 processors.

Tasks No.	U	U BOUND	EDF			LLF			EFSBA			OPBGA		
			DLM	ART	ATAT	DLM	ART	ATAT	DLM	ART	ATAT	DLM	ART	ATAT
5	0.12	0.743491775	0	0.063769411	0.401092905	0	0.056091052	0.393414546	0	0.007211601	0.344535095	0	0.007211601	0.344535095
20	0.12	0.705298477	0	0.079070851	0.126836433	0	0.087271362	0.135036944	0	0.058083376	0.105848957	0	0.044530864	0.092296445
35	0.12	0.70005633	0	0.094071399	0.133252342	0	0.075198615	0.114379559	0	0.050112386	0.08929333	0	0.036069251	0.075250194
65	0.36	0.696856145	2	0.605757461	0.660999505	0	0.351394287	0.406636331	0	0.15933479	0.214576833	0	0.095437929	0.150679972
65	0.6	0.696856145	0	0.283217809	0.390186556	6	1.221977668	1.328946415	0	0.973473081	1.080441828	0	0.175043507	0.282012254
50	0.84	0.69797399	1	0.80228782	0.975820215	1	0.848544358	1.022076753	0	0.347349779	0.520882173	0	0.268852549	0.442384943
65	0.84	0.696856145	1	0.811055191	0.949980032	2	1.072998142	1.211922983	0	0.622751021	0.761675862	0	0.262106056	0.401030897
80	0.84	0.696158703	4	0.945346238	1.06002278	1	0.92570498	1.040381522	0	0.438310891	0.552987433	0	0.212972585	0.327649127
65	1.08	0.696856145	5	1.639563581	1.805107746	4	1.225319209	1.390863374	0	0.4689597	0.634503865	0	0.310803265	0.476347429
50	1.32	0.69797399	6	2.36832419	2.725707252	3	1.353002803	1.710385865	0	0.427787308	0.78517037	0	0.423541064	0.780924126
65	1.32	0.696856145	2	0.812694373	0.997711792	6	1.077230642	1.262248061	0	0.516243231	0.70126065	0	0.325427165	0.510444583
65	1.56	0.696856145	3	1.186524176	1.490443359	9	2.002662778	2.306581961	0	0.846529004	1.150448188	0	0.475659209	0.779578392
80	1.56	0.696158703	2	1.095150745	1.320121854	3	1.490942924	1.715914034	0	0.821305583	1.046276693	0	0.451630407	0.676601517
65	1.8	0.696856145	4	1.488903342	1.820837543	9	2.445071958	2.777006159	0	0.974281758	1.306215959	0	0.504561335	0.836495536
80	1.8	0.696158703	2	1.854755266	2.086675816	3	2.368214285	2.600134835	0	0.783357755	1.015278304	0	0.436749283	0.668669833
65	2.04	0.696856145	11	3.944952116	4.35803717	5	2.177475186	2.590560241	0	1.475619901	1.888704956	0	0.624630461	1.037715516
80	2.04	0.696158703	4	1.708746028	1.902244378	9	2.421618801	2.615117151	0	1.546413487	1.739911837	0	0.478908031	0.672406381
50	2.28	0.69797399	2	1.934893814	2.16170488	3	1.453824409	1.680635475	1	1.110983038	1.337794104	0	0.69136774	0.918178806
95	2.28	0.695682042	15	3.217672224	3.530302023	8	2.773200059	3.085829858	0	1.278844359	1.591474158	0	0.554264618	0.866894416
50	2.52	0.69797399	4	3.302861572	3.870000033	5	2.933043647	3.500182108	1	1.134377707	1.701516168	0	0.866582652	1.433721113
110	2.52	0.695335652	6	2.182825005	2.365171446	10	2.366050959	2.5483974	1	1.52818799	1.710534431	0	0.551777668	0.734124109
80	2.76	0.696158703	10	3.072639058	3.562128936	15	3.69601465	4.185504528	1	1.130475038	1.619964916	0	0.731273599	1.220763477
110	2.76	0.695335652	20	3.166217608	3.525558266	22	4.395585198	4.754925855	0	1.148077189	1.507417846	0	0.580745878	0.940086536
80	3	0.696158703	1	1.256238347	1.62847212	19	5.902858802	6.275092574	3	2.243549002	2.615782774	1	0.791980072	1.164213844
110	3	0.695335652	2	1.041249451	1.407152115	12	2.574885112	2.940787776	21	4.60738417	4.973286833	0	0.659743689	1.025646353

In the instance with 10 processors, Table 5, the number of tasks ranged from 20 to 500. The load factors varied between 0.12 and 9.48, while the laxities ranged from zero to fifteen-time units. The deadlines were between zero- and thirty-time units.

**Table 5:** Performance measures in the case of 10 processors.

Tasks No.	U	U BOUND	EDF			LLF			EFSBA			OPBGA		
			DLM	ART	ATAT	DLM	ART	ATAT	DLM	ART	ATAT	DLM	ART	ATAT
40	0.12	0.699188	0	0.029183	0.086856	0	0.017571	0.075243	0	0.015204	0.072877	0	0.010463	0.068136
40	0.48	0.699188	0	0.084877	0.338442	0	0.08457	0.338135	0	0.058508	0.312073	0	0.010463	0.068136
60	0.84	0.697166	0	0.159764	0.378843	0	0.192478	0.411556	0	0.100069	0.319148	0	0.05851	0.277588
80	1.56	0.696159	1	0.480503	0.871951	0	0.418274	0.809721	0	0.206716	0.598164	0	0.108327	0.499774
100	1.92	0.695555	1	0.372835	0.527131	1	0.389567	0.543863	0	0.25449	0.408786	0	0.110922	0.265218
80	2.28	0.696159	1	0.950499	1.564171	0	0.495548	1.10922	0	0.356385	0.970057	0	0.179976	0.793649
100	2.28	0.695555	0	0.493606	0.842963	1	0.517324	0.866681	0	0.245247	0.594604	0	0.145091	0.494448
120	2.64	0.695153	1	0.535257	0.895983	2	0.458331	0.819057	0	0.30892	0.669646	0	0.146616	0.507342
120	3	0.695153	5	1.874167	2.251945	4	1.880486	2.258265	0	1.340186	1.717965	0	1.114533	1.492312
140	3	0.694866	7	2.004044	2.31639	7	2.101482	2.413827	0	1.35012	1.662465	0	1.077616	1.389961
160	3.72	0.694651	12	2.78756	3.168768	10	2.628651	3.009859	0	1.965818	2.347026	0	1.467634	1.848842
160	4.8	0.694651	12	3.069153	3.526045	12	3.114755	3.571647	0	2.210599	2.667491	0	1.850745	2.307637
180	4.8	0.694483	16	2.994135	3.361298	14	3.094565	3.461728	0	2.199649	2.566812	0	1.710411	2.077574
200	5.16	0.69435	23	3.210905	3.585946	22	3.514849	3.88989	0	2.28167	2.656711	0	1.828123	2.203164
180	5.52	0.694483	24	3.492801	3.935429	16	3.416528	3.859156	0	2.461495	2.904123	0	2.047936	2.490564
200	5.52	0.69435	26	4.330685	4.790153	15	4.093063	4.552531	0	2.822078	3.281545	0	2.215111	2.674578
240	6.6	0.694149	24	4.007802	4.391116	24	4.245141	4.628455	1	3.083016	3.46633	0	2.412678	2.795993
280	6.96	0.694006	41	4.842333	5.222687	42	4.715005	5.095359	1	3.468293	3.848647	0	2.666305	3.046659
280	7.32	0.694006	40	4.572831	4.94357	40	4.511101	4.88184	0	3.267783	3.638522	0	2.507267	2.878006
320	8.04	0.693898	47	5.694224	6.091569	53	6.251937	6.649282	0	4.064082	4.461426	0	3.174567	3.571911
400	8.76	0.693748	74	6.502477	6.844452	84	6.652556	6.994532	0	4.214921	4.556897	0	3.225951	3.567927
420	9.12	0.693719	79	6.202533	6.543746	83	6.669579	7.010791	1	4.316419	4.657631	0	3.520517	3.86173
420	9.48	0.693719	85	6.417867	6.763586	80	6.391364	6.737082	1	4.27154	4.617258	0	3.481468	3.827187
500	9.48	0.693628	111	6.725895	7.008608	109	6.56656	6.849273	0	4.253886	4.536599	0	3.306177	3.58889

Finally, for the one hundred processors scenario, Table 6, the number of tasks for each task set went from 100 to 3000, with load factors extending from 0.12 to 14.4. In this scenario, the value of deadlines went from one to fifty-time units, with laxities ranging from zero to twenty-time units.

**Table 6:** Performance measures in the case of 100 processors.

Tasks No.	U	U BOUND	EDF			LLF			EFSBA			OPBGA		
			DLM	ART	ATAT	DLM	ART	ATAT	DLM	ART	ATAT	DLM	ART	ATAT
500	0.72	0.693627856	0	0.051514446	0.087419855	0	0.051274626	0.087180035	0	0.041937662	0.077843071	0	0.035258077	0.071163486
700	1.32	0.693490475	0	0.107395275	0.15508306	0	0.104003189	0.151690974	0	0.080675275	0.12836306	0	0.060634959	0.108322744
500	1.92	0.693627856	0	0.149300035	0.252312635	0	0.154847125	0.257859724	0	0.114443575	0.217456174	0	0.089410285	0.192422884
500	2.52	0.693627856	0	0.187508354	0.31524602	0	0.170765416	0.298503082	0	0.135219156	0.262956823	0	0.112207841	0.239945508
700	3.12	0.693490475	0	0.253840162	0.365230837	0	0.273441176	0.384831851	0	0.207782904	0.319173579	0	0.150321785	0.26171246
700	3.72	0.693490475	0	0.315624099	0.448758288	0	0.323360014	0.456494202	0	0.235060953	0.368195142	0	0.177045098	0.310179287
900	4.32	0.693414167	0	0.383118555	0.501542357	0	0.388786518	0.507210321	0	0.276649813	0.395073616	0	0.205172925	0.323596727
900	4.92	0.693414167	0	0.433409439	0.568574545	0	0.426169361	0.561334467	0	0.339753994	0.4749191	0	0.258738519	0.393903625
900	5.52	0.693414167	3	0.503960492	0.663329796	4	0.493020309	0.652389612	0	0.374839825	0.534209128	0	0.272317787	0.431687091
1100	6.12	0.693365614	0	0.524714682	0.660175972	4	0.551263799	0.686725089	0	0.429525189	0.564986479	0	0.299015214	0.434476505
1100	6.72	0.693365614	6	0.578232949	0.732939562	4	0.598849513	0.753556125	0	0.442239736	0.596946349	0	0.32939387	0.484100482
1100	7.32	0.693365614	9	0.666411565	0.835887124	11	0.706180452	0.875656011	0	0.494849321	0.66432488	0	0.358042351	0.52751791
1300	7.92	0.693332003	6	0.709130521	0.857892159	12	0.739035479	0.887797116	0	0.535593381	0.684355018	0	0.384200435	0.532962073
1500	8.52	0.693307356	7	0.846762125	0.989374483	12	0.87768459	1.020296948	0	0.640015719	0.782628077	0	0.438170512	0.58078287
1700	9.12	0.693288509	7	0.878644335	1.011277296	12	0.88349456	1.016127521	0	0.668593131	0.801226092	0	0.454659865	0.587292826
1900	9.72	0.693273631	19	0.986592711	1.115231351	20	0.996909567	1.125548206	0	0.736356488	0.864995127	0	0.487249865	0.615888504
2100	10.32	0.693261587	27	1.071080635	1.198396925	24	1.097531868	1.224848158	0	0.790186304	0.917502594	0	0.518327585	0.645643875
2300	10.92	0.693251637	27	1.075495767	1.192307991	26	1.110234516	1.22704674	0	0.822208648	0.939020872	0	0.542688682	0.659500906
2300	11.52	0.693251637	29	1.177612223	1.30650551	39	1.231801647	1.360694934	0	0.872702263	1.00159555	0	0.616024311	0.744917598
2500	12.12	0.69324328	43	1.264015997	1.39015047	36	1.255784663	1.381919136	0	0.946617456	1.072751929	0	0.631371121	0.757505594
2700	12.72	0.693236161	49	1.367134989	1.488645928	52	1.392617054	1.514127993	0	1.010165979	1.131676918	0	0.652397048	0.773907987
2700	13.32	0.693236161	54	1.39925538	1.523785993	60	1.361595577	1.48612619	0	1.042652087	1.1671827	0	0.662545241	0.787075853
2900	13.32	0.693230024	47	1.430413841	1.550810853	56	1.408757814	1.529154825	0	1.043558757	1.163955768	0	0.677352535	0.797749546
2700	13.92	0.693236161	65	1.532750765	1.668012564	57	1.472302302	1.607564101	0	1.075055292	1.210317091	0	0.688480979	0.823742778
2900	13.92	0.693230024	63	1.505421098	1.628275828	67	1.479445637	1.602300366	0	1.082932847	1.205787577	0	0.682986168	0.805840898

The performance evaluation of four scheduling algorithms, Earliest Deadline First (EDF), Least Laxity First (LLF), Evolutionary Fuzzy-based Scheduling Algorithm (EFSBA), and Optimized Performance Based Genetic Algorithm (OPBGA), reveals valuable insights:

Deadline Compliance in all cases, OPBGA algorithm achieved a DLM value of 0, indicating that no task missed its deadline across various scenarios, showcasing their reliability in meeting task deadlines. However, EDF, LLF and EFSBA algorithms showed some missed deadlines in some cases. Whereas, Average Response Time (ART) in all cases, the OPBGA algorithm consistently achieved the lowest ART, indicating quicker task response times. EFSBA also delivers competitive response times. While, OPBGA consistently outperforms other algorithms in Average Turnaround Time (ATAT), emphasizing its ability to complete tasks swiftly. Again, EFSBA also performed well in this aspect. On the other hand, as the load factor (U) increased, the number of missed deadlines increased for EDF and LLF algorithms. However, EFSBA and OPBGA maintained a perfect record in all cases, showcasing their robustness in handling higher load factor.

## Conclusion

This research presented an Optimized Performance Based Genetic Algorithm (OPBGA) that works on “Soft Real-Time” scenarios on multiprocessor platforms and measure the differences between its results to those of the LLF, EDF and EFSBA approaches regarding DLMs, ART, and ATAT parameters. This technique is used to determine schedules having optimal as well as suboptimal lengths of time so as to achieve high performance. In accordance with the comparison, the OPBGA algorithm appears to be the most efficient and reliable across all cases, especially as the system load factor increases. It consistently achieved the lowest average response and turnaround times while maintaining a perfect record of meeting deadlines. EFSBA also performed well, particularly in low to moderate load scenarios. EDF and LLF are suitable for scenarios with lower load factors but may struggle to meet deadlines as the load factor increases. However, the choice of algorithm should consider the specific requirements and constraints of the system and its load factor. Processors are fixed in number, and they are all identical and execute independent and non-preemptive tasks. The proposed algorithm has limitations that need to be addressed. Assuming homogeneous processors and tasks may not reflect real-world heterogeneity. Additionally, using a MATLAB simulator may not capture real-world complexities, necessitating further validation in practical scenarios.

## Data Availability

The datasets analyzed and generated during this study are not available on any online platform. They were created using MATLAB, as mentioned in the manuscript, and any required portion can be obtained from the corresponding author upon reasonable request. Moreover, the authors are preparing a paper that will offer a benchmarking perspective on these research results. This paper will provide other researchers with a comprehensive understanding of this topic.This information has also been included in the manuscript.
